# "The Hospital" Nursing Mirror

**Published:** 1901-05-11

**Authors:** 


"The Hospital" Nursing Mirror.

Being the Nursing Section of "The Hospital."

[Contributions for this Section of "The Hospital" should be addressed to the Editor, "The Hospital" Nursing Mirror, 28 & 29 Southampton Street Strand, London, W.C.]

### Notes on News from the Nursing World.

### THE ENDOWMENT FUND OF QUEEN VICTORIA'S NURSES.

It is evident that every effort will be made to secure the success of the Women's Memorial to Queen Victoria. The committee of influential ladies who have charge of the movement, with power to add to their number, held a meeting last week, after the general meeting at Londonderry House, to consider in what form the resolutions passed with regard to the appeal to the women of the Empire for further funds to permanently endow Queen Victoria's Institute for Jubilee Nurses, should be carried out. It was decided that the wives of the Lord Lieutenants, and the mayoresses of the United Kingdom, be invited to identify themselves with the movement, and co-operate with Lady Londonderry's committee in carrying it out. In order that all classes might have the opportunity of participating, it was resolved that the country be systematically organised by counties and municipal boroughs, so as to ensure collecting cards finding their way to every village and remote hamlet. The lines of organisation are similar to those which were so successful in 1887. Subscriptions in that year were collected in sums from one penny upwards, the subscribers numbering between three and four million women. We see no reason why, with strong and representative local committees, which it is intended to call into being as soon as possible, the answer of this appeal should not be as satisfactory as that which followed the initiation of the "Women's Jubilee Offering," and resulted in £70,000 being handed over by Queen Victoria for the foundation of the great charity which it is now proposed to place on a more enduring basis, with a wider sphere of operations. At the present time the number of trained nurses at work is nearly 900, and the Council of the Jubilee Institute for Nurses are of opinion that 2,000 is the minimum supply necessary to justify them in regarding the Queen's wish as fairly established. This would mean the extension of the work to every town and district in the United Kingdom, and the creation of a permanent memorial of Queen Victoria at the door of all the poor whose sufferings she was always so deeply concerned to alleviate.

### FOR SOUTH AFRICA.

We are informed by the Secretary of State for War that the following nurses embarked for South Africa on Saturday last on the hospital ship *Nubia*:—Misses M. Atkinson, B. Batty, A. M. Beale, A. S. Bond, L. Dale, A. Guthrie, V. P. Squire, and Mrs. Bourchier. Miss Annie Mildred Beale was trained at St. Mary's Hospital, Paddington, where, after her training, she was night and staff nurse for nearly two years. Since September, 1896, she has been engaged in private nursing.

### THE SISTERS AND THE SOLDIERS' GRAVES.

A correspondent of the *Johannesburg Gazette*, of April 10, writes:—"I happened to go to the cemetery at Krugersdorp on Easter Day morning to see how our poor Tommies' graves are kept, and I can tell you I was surprised to find them all beautifully decorated with fresh flowers, simply magnificent wreaths, crosses, bouquets, &c., of fresh roses, violets, and other flowers. I made inquiries as to who performed this Easter service for our poor fellows, and found that the chief worker was Nursing Sister Yeoman, of No. 1 Stationary Hospital, assisted by Nursing Sister Smallman and Mrs. Capt. Stonestreet. There are 82 graves, including four Jameson Raid troopers, and all were equally beautifully tended. I think it would be some comfort to the friends of the poor fellows to know that even in this far-off land the graves of those dear to them are well tended and cared for, and some encouragement to the good women who tend them so lovingly."

### THE NURSING QUESTION IN IRISH POOR LAW INFIRMARIES.

We are officially informed that on Tuesday next the Order of the Local Government Board for Ireland, dated February 4th, 1901, will be appealed against at the Privy Council Chamber, Dublin Castle, by numerous Boards of Guardians under Section 14 of 10 & 11 Victoria, cap. 90. As our readers are aware, the question involved is the right of the Local Government Board for Ireland to insist upon the appointment of qualified nurses and attendants in Poor Law Infirmaries and Hospitals. The issue involved is one of great importance, for the Order applies to no less than 159 Unions in the sister island.

### AN ARMY CHAPLAIN ON ARMY SISTERS.

At the annual meeting of the Bangor Nursing Institution the Rev. Frank Edwards, who has been engaged as chaplain to the forces in South Africa, paid a striking tribute to the Army nurses. Speaking from personal experience, Mr. Edwards remarked that he could not dilate too highly on their work.

Their patient devotion to duty, their self-sacrifice, their cheerfulness and courage amidst peril, privation, and hardship, had been as heroic as many a gallant deed which had won the Victoria Cross. What had impressed him during his twelve months' experience in South Africa was what he might term the grit with which the nursing sisters had stuck to their work in the midst of most distressing surroundings and trying conditions.

Mr. Edwards having told his audience that he had seen nurses at work in the permanent hospitals, the field hospitals, on the march, hastening to the front in open railway trucks, exposed by day to the heat of a burning sun and by night to a pitiless wind, battling in the darkness against wind and rain, in some cases the night being so dark that the flashes of lightning were the only guidance they had to enable them to reach the tents where their services were required, faring cheerfully on the rations of the soldiers when the men themselves were complaining of the fare supplied to them, mentioned the case of a nurse who left Kroonstadt in an open cart, "her only escort across the veldt through the enemy's lines being a Kaffir boy." He was proud to say that that nurse was a native of Bangor. At Kroonstadt, Johannesburg,

Pretoria, Bloemfontein, Ladysmith, and Kimberley were the graves of nurses who had died in the performance of their duty.

### A DEVOTED MATRON.

The late Miss Ellen Kayser, who had been matron at the Plague Hospital, Maitland, since it was first started, was a member of the Victoria Nurses' Institute, Capetown, and her death is a serious loss to that organisation. Miss Dawson, the matron of the Institute, writes us that Miss Kayser's devotion to her work at the Plague Hospital was unceasing—" she never appeared to have a thought of self, and even in her dying moments her great anxiety seemed to be that those nursing her should not be overdone."

### THE NOTTINGHAMSHIRE NURSES.

Among the towns which show a large increase of population since 1891 is Nottingham. The number has risen in ten years from 213,877 to 239,753. It is, therefore, not surprising that at the annual meeting of the Nottinghamshire District Nursing Association, which has now existed for a quarter of a century, attention was specially drawn to the portion of the report that emphasises the growth of the work in the last ten years. During that period the number of patients requiring the nurses' services has more than trebled, and the actual amount of work done has nearly doubled. In the past year 943 cases were attended and 30,482 visits paid, as against 309 cases and 16,815 visits in 1890. This is in spite of the fact that 1900 was a year notable for the freedom of the town from illness; and the twelve nurses who now constitute the staff are not therefore sufficient. Happily, Sir Charles Seely is going, not for the first time, to render material assistance by the erection of a house for the nurse in one of the districts. But there has been a decrease of the funds, and the Association have had to draw upon their capital to the extent of £200. The Mayor, who presided at the meeting, expressed his pleasure that the contributions of poor patients, after recovery, amounted to nearly £16; and warmly eulogised the work of Miss Forrest, the late lady superintendent, who died two months after her resignation.

### A SHRINKAGE AT ABERDEEN.

At the annual meeting of the Aberdeen District Nursing Association the chairman, in moving the adoption of the report, had to express his regret that though the income was upwards of £600, there was a balance on the wrong side of £74. He attributed this shrinkage in the contributions to the excessive drain made upon the charitable public for national objects. A satisfactory feature of the position is that the artisans whom the association mainly benefits largely support it. As to the work of the nurses, upwards of 22,000 visits were paid last year, and 731 cases were nursed. The association has now existed for ten years, and it has steadily increased in vigour. The branch at Woodside has not only succeeded in paying its way, but the income exceeds the expenditure.

### MISS BAXTER'S RESIGNATION.

A full account of the annual general meeting of subscribers to and friends of the County and City of Cork Hospital for Women and Children, has been forwarded to us, and after a careful perusal of the proceedings we shall be surprised if the feeling of indignation which prevailed in Cork as to Miss Baxter's resignation does not quietly subside. The Bishop of Cork, who was in the chair, Sir George Colthurst, and Mr. L. A. Beamish, highly eulogised the late lady superintendent for the manner in which she discharged her duties, and a paragraph of the report of the committee was as follows :—

> Miss Baxter has for the past fourteen years acted as lady superintendent, and the board would like to place on record their full sense of the good work she has done during these years, and the high state of perfection to which she has brought the hospital by her constant care and attention. The board regret that she has not been able to see her way to continue as lady superintendent, owing to the changes that they have been compelled to adopt.

The general and personal tributes to the services of Miss Baxter should do much to remove the impression that her work has been overlooked, though perhaps the expression of public opinion in Cork has something to do with the tone of the speakers at the meeting and the votes of thanks which were accorded to her. As to the cause of Miss Baxter's retirement, the treasurer put the case very shortly. He said that owing to the need of avoiding a state of insolvency the Board of Management were called upon to act more stringently than they had done in the past in carrying out the rules of the hospital, and Miss Baxter did not feel that it would be possible for her in the future to submit herself to these rules. The committee had, in fact, to choose between carrying out their scheme of reform and her resignation, and they reluctantly preferred the latter. We do not know why the laxity now confessed with regard to the rules was permitted in the past, but it is a sound principle that no official, however able and devoted, shall be allowed to assume the position of dictator.

### THE ADVERTISEMENT THAT FAILED.

A superintendent of nurses has been appointed by the Croydon Board of Guardians, but from the information supplied to us, we gather that the attempt to secure the services of a lady doctor willing to assume nursing responsibility, who would thus be a link between the medical and the nursing professions, has failed. The new official, whose training is not stated, is now nursing lecturer under the London School Board, and has been lecturer on nursing and hygiene for the Northampton County Council, superintendent of nurses at the Tower Hamlets Dispensary, and nurse-matron of the Stepney Nursing Home. It will be observed that, according to the official intimation, she has had neither hospital nor infirmary experience.

### THE NURSES OF BIRKENHEAD HOSPITAL.

An appeal for funds has been issued by the Committee of the Birkenhead Borough Hospital. The most urgent need appears to be a nurses' home. At present the nurses are provided with what a daily local paper calls "lodgment in a cellar," and it is recognised that they require " better accommodation." There is some property close to the hospital which could easily be adapted for use as a nurses' home, and as it belongs to the Corporation, the suggestion is made that it should be sold to the hospital committee on reasonable terms. It is estimated that about £4,000 is wanted in order to carry out the improvement scheme. It can scarcely be doubted that the sum will be forthcoming; Birkenhead is too important a town to remain under the reproach that the hospital nurses live and sleep in a cellar.

### GLASGOW MATERNITY HOSPITAL.

In the sixty-sixth annual report of the Glasgow Maternity Hospital it is stated that the number of nurses who are trained annually is well maintained. From the medical report we learn that 2,831 cases were attended in the various departments of the hospital, the number in the indoor department being 602. This was an increase of 88 over last year, and by far the largest number ever treated in one year. It speaks well for both the work of the medical and the nursing staff that, though in consequence of the number of patients being so large both flats have frequently been occupied simultaneously, the general health of the patients has never been better.

### A START AT CLAY CROSS.

It has been decided to start a nursing institution at Clay Cross, and we learn with interest that the first movement was made by the Friendly Societies' Council in the town, who convened a representative meeting for the purpose of ventilating the subject and forming a nucleus for the proposed organisation. Subsequently a concert for the benefit of the fund was given, and another meeting has since been called by the Clay Cross Urban Council, at which it was unanimously decided that the institution should be a local memorial to Queen Victoria. Dr. Duncan, who was one of the speakers, said he had been struck with the thought that a nurse was an absolute necessity for the place, and it was a wonder to him that one had not been appointed before now. It is calculated that about £100 will be required for the first year, and the Vicar of Clay Cross, in reading out the terms of the appeal to the townspeople, emphasised the need for regular subscriptions to maintain the institution. This is always a vital point, and it is wise to give it prominence at the outset.

### QUEEN'S NURSES AT ROCHDALE.

It is proposed to erect a new nurses' home at Rochdale. The existing one is only just large enough for the present staff. At the quarterly meeting of the District Nursing Association, which is affiliated to Queen Victoria's Jubilee Institute, it was stated that last year 493 cases were nursed and 9,912 visits were paid. The Association has four nurses at work in Rochdale, and one in Castleton. As the visits paid in the quarter ending March 31st were 3,606, as compared with 2,277 in the same period last year—the cases being 152 as against 112—it is clear that if the sick poor are to receive proper attention the number of nurses must be augmented. It is true that the funds of the Association were not sufficient in 1900 to meet the expenditure; but Rochdale is only one of many places which have suffered from the urgency of exceptional calls on public liberality, and if the nurses of the town are to do their work efficiently, they must be provided with adequate accommodation.

### REIGATE ISOLATION HOSPITAL.

A correspondent, who should be well-informed, flatly contradicts the statement in our last issue that Miss Dundas has been appointed matron of Reigate Isolation Hospital, and declares "that neither Miss Dundas, the doctor, nor the committee know anything about the intimation sent to '*The Hospital*' *Nursing Mirror*." What, then, is the meaning of the official announcement in the *Reigate Borough Advertiser* for April 30th, according to which the sanitary committee of the Town Council reported that "Miss Dundas was appointed matron in the place of Miss Amcoats at £40 a year." Judging by the remarks of the author of a letter in another column, the position of affairs at the Reigate Isolation Hospital is unsatisfactory. The writer, we observe, says that the sanitary inspector, in spite of the prohibition of the Town Council, continues to exercise control over the hospital staff. But surely the Council has the power to enforce its own decrees. We certainly do not think that it is a suitable arrangement for a sanitary inspector to be put over the doctor and matron of any hospital.

### THE PIANO AT CARDIFF.

The Cardiff Board of Guardians have been discussing the question of providing, or assisting to provide, a piano for the use of the nurses in their employ. The nurses have subscribed £13 towards the value of the instrument, and they asked the Guardians to give £20 in order to complete the purchase, on the understanding that the piano should become the property of the Board. The matter has been referred to a committee, and meanwhile an effort has been made by a local paper to deride the proposal and ridicule the Guardians by saying that they contemplate buying pianos for the paupers. This is unfair; but we hope that such misrepresentations will not hinder the Board from making a concession which adds a little brightness to the lives of the workhouse infirmary nurses.

### AN EXPERIMENT AT EXETER.

In engaging an assistant nurse, the St. Thomas's Board of Guardians have arranged for her to undertake night duty only, and to reside out of the house. While approving of the appointment, the Local Government Board have signified disapproval of the principle of employing an exclusively night nurse, and also of the assistant living out. They are, however, willing that the arrangement should be tried for three months, and they have requested to be informed at the end of that period how it works. The reason for the residence out of the house is that the accommodation inside is not sufficient.

### ONE WAY OF HELPING NURSING ASSOCIATIONS.

A singularly successful concert has been given at Coalville, in Leicestershire, on behalf of the Nursing Association. It was promoted by the Coalville and District Trades Council, who twelve months ago got the choir together in order to make the entertainment as attractive as possible. It is not, of course, unusual to organise a concert in aid of a nursing association—two concerts of a very excellent character have just been given at Romsey on behalf of the Jubilee Nursing Home—but it is particularly gratifying when practical evidence of the gratitude of such a body as the Coalville Trades Council for the advantages their members derive from the Nursing Association is forthcoming.

### SHORT ITEMS.

The balance (£15) of the amount subscribed by the Queen's Nurses throughout England, Scotland, Ireland, and Wales for a wreath in memory of Her late Majesty Queen Victoria, has been sent to the hon. treasurer of the Queen's Nurses' Endowment Fund as a "donation to the Fund from the Queen's Nurses."—On behalf of the Royal Albert Hospital, Devonport, a very brilliant bazaar was held last week in the Public Hall. Quite a feature of the show was the hospital medical staff stall, surmounted by the Red Cross of Geneva, wreathed with ivy, and full of saleable goods. The matron and sisters were in charge, and were assisted by a number of Devonport ladies.—The late Sir Edwin Saunders has bequeated £100 to the Royal British Nurses' Association.

### Lectures to Nurses on Medicine and Medical Nursing.

By NORMAN DALTON, M.D. London, F.R.C.P., Physician to King's College Hospital.

### LECTURE X.—HEART DISEASE: BACK-PRESSURE—COMPENSATION.

*Heart Disease.*—This usually means disease of the valves, but, in a few cases, the muscular wall of the heart is so weakened by fatty degeneration that it cannot keep up the circulation of the blood although the valves are healthy. In such cases the same symptoms may occur as when the valves are diseased. The commonest cause of valvular disease is inflammation of the endocardium (endocarditis), which is the membrane which lines the inside of the heart, including the valves. In its turn the great cause of acute endocarditis is rheumatic fever. Another cause is septicæmia, while scarlet fever and the other fevers may possibly be causes. In some cases no cause can be discovered, but recent researches show that these cases may really be due to the poison of rheumatic fever, which, instead of causing inflammation of the joints as it usually does, has produced tonsillitis, St. Vitus' dance, &c., but has, nevertheless, affected the heart, just as in cases of rheumatic fever. Some cases of endocarditis are chronic from the first and come on insidiously. These may be due to gout, syphilis, alcohol, &c.

The valves of the heart are thin, flexible, and so shaped that when they come together, they fit accurately and close tightly the orifice which they guard. On the other hand, when the blood is passing through these orifices in the right direction they flatten themselves back against the wall of the heart or the aorta and offer no obstruction to its passage. The effect of endocarditis is to make the valves thick, rigid, and irregular in shape. Consequently when they come together they do not fit accurately and do not completely close the orifice which they guard. Also they may stand out stiffly and narrow this orifice, so that the blood may not pass freely through it.

If the mitral valves cannot close the mitral orifice, the blood is thrown back into the left auricle when the left ventricle contracts. This is called "mitral regurgitation." If the mitral orifice be obstructed by the rigidity of its valves, the blood cannot pass freely into the left ventricle when the auricle contracts. This is called "mitral stenosis." In "aortic regurgitation" the blood drops back from the aorta into the left ventricle as soon as this ventricle has finished contracting, and in "aortic stenosis" the blood cannot pass freely through the aortic orifice because that orifice is narrowed by the rigid, projecting valves. Regurgitation and stenosis may also occur at the orifices in the right side of the heart, but this is rare.

It must be noticed that the effect of regurgitation and of stenosis on the circulation is much the same. If we take the mitral valve as an example, it is obvious that in both regurgitation and stenosis the left auricle must become over-distended; for in the case of regurgitation, no sooner does the left auricle empty itself into the ventricle than the ventricle throws the blood back into the auricle through the unclosed mitral orifice. Now at this time the blood is pouring into the auricle from the pulmonary veins so that the auricle must become over-distended. In mitral stenosis, on the other hand, the auricle can never empty itself completely because the narrow orifice will not let the blood pass freely into the ventricle, and as the blood continues to enter the auricle from the lungs, over-distension must result. In many cases both regurgitation and stenosis take place at the same valve because the valves are distorted and rigid and will neither shut nor open completely.

In all that has been said, and that is going to be said, it is assumed that nurses have a knowledge of the main facts about the circulation of the blood. Having shown that in disease of the mitral valve, the left auricle is habitually over-distended, it must be pointed out that this over-distension makes it difficult for the blood which comes from the lungs to enter the auricle. Consequently the lungs become congested; that is, their capillaries become over-distended with blood. This in time make it difficult for the right ventricle to pump the blood through the lungs, and for the right auricle to pump the blood into the right ventricle, and for the blood in the venæ cavæ to run into the right auricle. Now the circulation in the veins of the face and arms is helped by the force of gravity, so that the blood does pass fairly well from them through the superior vena cava into the right auricle, although the lips and hands are often somewhat cyanosed. But the force of gravity is against the circulation of the blood through the veins which run into the inferior vena cava, so that as soon as the difficulty in entering the right auricle arises the blood has great difficulty in getting through them and their capillaries become overdistended with blood; this means that the liver, kidneys and legs, &c., become congested. When the congestion has lasted some time dropsy sets in in the congested parts. This does not occur to an appreciable extent in solid organs like the liver and kidneys; but the legs, the serous cavities, and the lungs become filled with serum, as was explained in the lecture on dropsy.

The over-distension of the venous system and consequent congestion of the organs which has just been described, constitute what are called the "back-pressure effects of valvular disease of the heart," and the symptoms which result from the congestion of the organs, are called "back-pressure" symptoms.

In persons who are otherwise healthy, a slight defect of the valves may be so completely overcome by "hypertrophy of the heart" that no back-pressure or other symptoms arise. In "hypertrophy" new muscular fibres grow in the wall of the heart, so that it becomes thicker and stronger; and, at the same time, the cavities of the heart become a little bigger, so that they hold more blood. It will be obvious that in the case of the mitral valve, if the left auricle be stronger than normal, it may succeed at each contraction in getting a normal quantity of blood through the orifice of the valve, although the latter be narrowed, and that in regurgitation, if hypertrophy be present the auricle will be large enough to hold comfortably both the blood coming from the lungs and that which comes back from the left ventricle and will be strong enough to pump all of it into the ventricle. Hence no blood stays in the auricle, and no back-pressure takes place. The hypertrophy, therefore, completely compensates for the valvular defect, and when hypertrophy is sufficiently good to prevent back-pressure symptoms, "compensation for a valvular defect" is said to be established. It follows that when back pressure symptoms occur, we know that compensatory hypertrophy is ncomplete.

In "dilatation" of the heart, its cavities become larger without any growth of new muscular fibres in their walls. Consequently the walls are thinner and their expulsive force weaker, so that once dilatation has set in, back-pressure symptoms soon follow.

### To Nurses.

**WE invite contributions from any of our readers, and shall be glad to pay for "Notes on News from the Nursing World," or for articles describing nursing experiences, or dealing with any nursing question from an original point of view. The minimum payment for contributions is 5s., but we welcome interesting contributions of a column, or a page, in length. It may be added that notices of entertainments, presentations, and deaths are not paid for, but, of course, we are always glad to receive them. All rejected manuscripts are returned in due course, and all payments for manuscripts used are made as early as possible after the beginning of each quarter.**

### Across the Seas.

### NURSING AT WINNIPEG GENERAL HOSPITAL.

By a Graduate of 1899.

*(Concluded from page 55.)*

On January 1st I was transferred to the general wards, and became third nurse in the women's surgical ward, where I stayed for two months on day duty. My next move was to the men's medical ward on night duty. The cases were chiefly typhoid, which was almost epidemic in the city at that time, and the hospital had to accommodate far more patients than it was meant for. The night duty was arranged in such a way that a pupil nurse of some months' standing was in charge of each public ward, whilst a senior pupil nurse looked after the private ward flat, containing seven private wards. A night supervisor, who was either a nurse in her third year or a graduate, superintended the whole hospital at night, giving help in any ward where she was needed, but having her headquarters on the Ward II. flat, where she had two private wards and a small surgical ward to attend to herself. About ten o'clock we had our first meal—just a cup of tea with bread and butter and cake in the ward kitchen. The lady superintendent always came over from the Nurses' Home to share it with us. Very often we had hardly time to sit down for it, and took a hasty cup of tea standing up; but on peaceful nights, when the ward was not too busy, we had a quarter of an hour for a little chat and to have our fortunes read in our teacups. Do nurses all over the world read fortunes in tea-leaves? It was our pet amusement, and no nurse could ever hope to be really popular unless she could see romantic and dream-like visions in a prosaic cup of hospital tea.

### On Night Duty.

I think it was during my night duty in Ward II. that for the first time I learnt self-reliance. The ward was filled with critical cases, and I was often left for a long time by myself, the night supervisor having already more than enough to do in her own wards and the rest of the hospital. So I began to find that I had to look to myself to recognise dangerous symptoms, know when it was necessary to call a doctor, and, above all, when it was not necessary; to act quickly in emergencies and to keep my head cool and my wits collected under the most trying circumstances. My patients, as a rule, were very amenable to discipline, and gave me very little trouble in that way: they seemed to think that I had far too much to do; and, indeed, I found that night duty very hard work. Ice-baths and sponging added to the two-hour and four-hour charts and the endless amount of treatment and care which typhoids alway require, kept me busy for the twelve hours of duty. But in spite of the hard work I always look back on those "rushing" months as some of the happiest days I spent in the hospital. I am afraid that it caused me unspeakable joy one day to hear a very sick but quite sensible C.P.R. fireman remark to a typhoid in the next bed, "Talk about those day nurses, why the night nurse is worth six of them any day. Anyway it takes six of them to run the ward in the day time, and only her on at night." The fact that any one of the day nurses would probably have been able to do exactly the same did not detract at all from my pleasure at hearing that little aside.

### Back to the Isolated Block.

I remained on night duty for four months, having learnt many things in that time, not only in the way of actual nursing, but also as to the management of a ward and the individual characteristics of all sorts and conditions of patients, and I was heartily sorry when I was transferred in August to day duty in Ward III. again. However, it was only for a short time for at the end of a week I was informed that the "Isolated" was again to be my destination, and after having a whole day off duty, I went over to Section F to nurse diphtheria. I found the work when I first went over very light: four convalescents waiting for a third negative report from the laboratory before being allowed to return home and a fellow nurse who had contracted the disease whilst on duty in the section, were my patients. Of course I was not allowed to take my books into the wards, but when my work was finished, and my patients were resting, I used to sit on the verandah which overlooked the prettiest part of the garden and sew or study. An outside staircase was the only means of reaching the diphtheria section, which was on the second floor of the Isolated building, and it was sometimes very trying in the middle of a Canadian winter, with the thermometer at 4° below zero, and perhaps a blizzard blowing, to make one's way up those long steps covered with snow and ice. I believe that a nurse in desperation came down in the little lift which conveyed the food from the kitchen to the ward, but the experience was not pleasant enough to tempt anyone else to follow her example.

### The Prevention of Contagion.

Our rules for the prevention of contagion were stringent. One particular regulation which we deplored and groaned over was that we were not allowed to wear our uniforms to or from the home to the hospital. On getting up in the morning we put on a cotton dress which we wore over to the hospital, and after having had breakfast in the downstairs dining-room we went to our respective wards and changed into uniform. To go down to meals we were provided with a large gown of brown holland, which was put on over the whole uniform and worn while we were downstairs. Any departure from this rule always met with a reprimand from the head nurse, or anyone in authority who happened to catch us out not properly attired. Many patients came in and went out, but I had only three really serious cases of diphtheria. The others were of a mild type and soon recovered, the patients all being treated with antitoxin almost as a matter of routine.

### How I Occupied Spare Time.

Some of my spare time I spent in making a very gorgeous screen for the amusement of my little patients, who were chiefly small children between the ages of two and seven years. The screen was divided into three folds, and each fold I covered with a different class of pictures, the first being devoted to the sea, with coloured pictures of ships, sailors, fishermen, &c.; the second was covered with brilliant pages from the fairy books, wild animals and strange birds and beasts; while the third was composed of Christmas cards, nursery-rhyme pictures, and other home-like scraps. In the middle of the fairy-picture fold was a large and fierce-looking tiger, which I often described to my little patients in the little verse beginning—

> "Tiger, tiger, burning bright,
> In the forests of the night."

Much to my surprise one day the smallest boy in the ward, on being asked by the visiting doctor what the great big animal on the screen was, answered glibly, "It's a tiger, tiger, burning bright, in the forests of the night."

### A Holiday.

I spent two months in Section F, and then, after disinfecting myself from head to foot in I don't know what strength of perchloride solution, I went home for a holiday. I had not taken the two weeks which were due to me at the end of a year, so that when eighteen months had passed I was allowed to be away for a whole month. How I enjoyed that holiday! Everything seemed delightful to me. I particularly appreciated not having to get up at 6 A.M. every morning. My holiday seemed only too short, for as I had to spend three days on the train each way I really only had three clear weeks at home. But, though I was sorry to leave again so soon, I returned to the hospital with very different feelings from those with which I had entered it, and looked forward with pleasure to my second year and a half in uniform.

### The Metropolitan Asylums Board and their Matrons.

BY OUR OWN CORRESPONDENT.

AT a meeting of the Metropolitan Asylums Board on Saturday, a letter was read from the Local Government Board confirming the appointment of Miss Rose Turner, L.R.C.P., L.R.C.S. Edin., L.F.P.S. Glas., medical attendant at the school for defective children, Elm Grove, Peckham, until March 25th, 1902.

On the recommendation of the children's committee it was decided that the annual leave of matrons at the seaside homes and the homes for defective children should be four weeks. Under the scale of uniforms for female staff at the hospitals of the Board, the issue of belts and shoes to nurses is limited to one belt and one pair of shoes for each nurse half-yearly. This number, the hospitals committee reported, was in practice found inadequate, and they therefore recommended: That the uniform scale in respect of female staff at the hospitals be amended, so as to permit of four belts and three pairs of shoes per annum being issued to each member of the nursing staff, if and when in the opinion of the matron concerned such numbers of articles are required. The recommendation was adopted.

The hospitals committee reported that they had received thirty-four applications for the matronships of the Park and the Fountain Hospitals, and they recommended the appointment of (*a*) Miss Annie Thomas to the Park Hospital on probation for a period not exceeding three months. Miss Thomas has for the past twenty months held the post of assistant matron at the Board's Grove Hospital. She formerly held the office of staff nurse at the Birmingham General Hospital, and before her appointment as assistant matron of the Grove Hospital, was charge nurse and night superintendent at the Brook Hospital for one year and seven months. (*b*) Miss Susan Alice Villiers, to the Fountain Hospital. Miss Villiers has for over two years held the office of assistant matron at the Brook Hospital. She formerly held the office of probationer and staff nurse at St. Bartholomew's Hospital for four years, and was then appointed superintendent of night nurses at the South-Eastern Hospital for three years.

Mr. J. H. Lile moved that recommendation (*a*) be referred back. In doing so he protested against the way in which Miss Atkins, a former matron of the Grove Hospital, had been treated. Miss Atkins, he stated, some twelve months ago, applied to the Board for leave of absence, in order to go to South Africa to nurse the fever patients in the military hospitals at Capetown. The Board refused to grant the leave of absence, and Miss Atkins resigned her appointment of matron of the Grove Hospital, and went to Capetown. It was, he added, generally understood at the time that "when the war was over" she should be considered in the event of the matronship of one of their hospitals becoming vacant. He thought that the patriotic action of Miss Atkins in giving up her position and going to the front to nurse our fever-stricken soldiers should highly commend itself to the Board.

Captain Andrew seconded the amendment.

Mr. A. C. Scovell, Chairman of the Hospitals Committee, doubted very much whether Miss Atkins would be prepared to accept an appointment under the Board. She was at present employed by the Government in nursing the sick and wounded on the transports from the Cape to Southampton. She had, he understood, a very agreeable position, and enjoyed a salary of £150 per annum, which was more than she would receive as matron of one of their hospitals.

On being put to the vote the amendment was lost, and the report, together with the two recommendations, were adopted.

### Appointments.

BURNLEY UNION WORKHOUSE INFIRMARY.—Miss Bridget Nugent has been appointed superintendent nurse. She was trained at the Birmingham Infirmary. Since her three years' training she has been sister successively of a male surgical ward, sister of a female surgical ward, and sister in charge of the lying-in block in the same institution. Miss Nugent holds the L.O.S. certificate.

CROYDON UNION INFIRMARY.—Mrs. D. Coburn-Coulton has been appointed superintendent of nurses. She is now nursing lecturer under the London School Board, and was previously lecturer on nursing and hygiene for the Northampton County Council, superintendent of nurses at the Tower Hamlets Dispensary, and nurse matron of the Nursing Home at Stepney.

DEAN CLOSE SCHOOL SANATORIUM, CHELTENHAM.—Miss Alice E. Judd has been appointed resident nurse. She was trained at the London Hospital, and was subsequently staff nurse, and a member of the private staff. She has since been attached to the Nurses' Co-operation, has done district work at Ashton-on-Ribble, St. Leonards, and in London. Miss Judd holds one of the medals awarded in connection with the typhoid fever epidemic at Maidstone.

FOUNTAIN HOSPITAL, TOOTING GRAVENEY, S.W.—Miss S. A. Villiers has been appointed matron. She was trained at St. Bartholomew's Hospital, and has since been night superintendent at the South-Eastern Hospital, New Cross, and assistant matron at the Brook Hospital, Shooter's Hill, under the Metropolitan Asylums Board.

MARKET DRAYTON COTTAGE HOSPITAL.—Miss Rose has been appointed matron. She was trained at the Walsall and District Hospital, and has since been sister for six years at the Guest Hospital, Dudley, sister at the Royal Eye Hospital, Manchester, sister of the men's surgical ward at the General Hospital, Wolverhampton, and night sister at the County Hospital, York.

MEMORIAL COTTAGE HOSPITAL, LEEK.—Miss M. Lamble has been appointed matron-nurse. She was trained at North Staffordshire Infirmary and Eye Hospital, and has since been charge nurse at North Staffordshire Infirmary, sister at the Ulster Hospital for Women and Children, Belfast, sister and night superintendent at the Guest Hospital, Dudley, and sister at the Royal Berks Hospital, Reading.

PARK HOSPITAL, HITHER GREEN, S.E.—Miss A. Thomas has been appointed matron. She was trained at the General Hospital, Birmingham, and has since been charge nurse and night superintendent at the Brook Hospital, Shooter's Hill, and is assistant matron at the Grove Hospital, Lower Tooting, S.W., under the Metropolitan Asylums Board.

STAPLETON UNION HOSPITAL.—Miss E. S. Owen has been appointed superintendent. She was trained at the Liverpool Royal Infirmary, where she remained as staff nurse for some years. She has since been charge nurse at Toxteth Park Union Hospital, Liverpool, and at the Union Infirmary, Fir Vale, Sheffield, superintendent nurse at Luton Infirmary, and superintendent nurse at Stepney Union Workhouse. She holds the L.O.S. certificate.

WEST HAM HOSPITAL, STRATFORD, E.—Miss Annie Nutter has been appointed staff nurse. She was trained at Leeds Union Infirmary, where she was subsequently staff nurse for a time. She has since been charge nurse at Prescot Union Infirmary, and staff nurse for male wards, theatre, and receiving room at West Ham Hospital.

### Presentations.

STAMFORD NURSING SOCIETY.—Last week the committee of the Stamford Nursing Society presented Nurse Edith Hughes, who is leaving to take up similar duties in Northampton, with a purse of money valued £10. This amount had been collected by the Mayoress (Mrs. C. Gray) and other ladies, and the presentation was made by Alderman Knott. Miss Hughes suitably acknowledged the gift.

### Echoes from the Outside World.

### AN OPEN LETTER TO A HOSPITAL NURSE.

A CORRESPONDENT from Natal refers to overcrowded steamers coming to England. The continued epidemic of illness, which is, I suppose, only the natural result of the war, has very much depressed the colonists, already sad at heart with loss of friends and property and with prospective famine staring them in the face. Therefore, with fever, either enteric or dengue in nearly every house, and the ever-present fear that the bubonic plague may spread inland from Capetown at any time, those who can do so are taking a trip to England for rest and refreshment. Even the prospect of seeing the Duke and Duchess of Cornwall and York a little later on does not appear to offer sufficient attractions to detain them, though many preparations are in hand, I hear, to give them a royal welcome. The Loyal Women's Guild is most busy trying to get together £5,000 as a foundation capital to found a scholarship for South African girls in memory of Queen Victoria. The women of Durban have also collected £500 for a painting of the Queen to be unveiled by the Duchess of York, and there is yet another scheme to build a convalescent home somewhere on the high healthy land beyond Durban for poor women as an additional memorial of the great and good Queen. The need of it is said to be extreme, because Durban does not become more healthy each year, and it is almost impossible for the toilers to go on month after month with no change and avoid running serious risks of permanently losing their health. Three commemoration schemes for one small colony which has already been almost devastated of its wealth are rather wonderful, I think, and highly significant of its loyalty.

FIRST impressions of this year's Academy naturally carry one's thoughts back to the sad days between January 21st and February 2nd. Someone has spoken of the exhibition at Burlington House as "In Memoriam," and though it is hardly that, the shadow of bereavement so persistently hangs over the gallery that from first to last the eye is unconsciously looking out for fresh glimpses of Victoria and Victoria's greatness. Ascending the staircase, we come face to face with a colossal seated statue of the late Queen, which has been designed by Mr. Onslow Ford, and is to be executed in marble and bronze for erection in the city of Manchester. The situation, of necessity, is so cramped that it is a little difficult to judge of the merits of the statue, which as it is seems to be unduly wide. This apparent defect will probably disappear when the statue is placed upon a large site. In the Lecture Room is another piece of sculpture—the Queen as she appeared twelve years ago, and naturally one gazes with additional interest upon the bust which is very fine both as a work of art and as a portrait, remembering that the memorial statue to be placed near Buckingham Palace has been entrusted to this artist, Mr. T. Brock. In the Fourth Gallery Mr. William Wyllie has given a representation of the funeral *cortège* crossing from the Isle of Wight to the mainland. Those who saw the procession will at once remark that the distance between the ships is much less in the picture than it appeared in reality, but that must be forgiven the artist, who had only a limited canvas at his disposal, and the "Passing of a Great Queen" is one of the best pictures Mr. Wyllie has painted. There is also a representation of the funeral procession as it passed up St. James' Street, called "2nd February, 1901," at which the painter, Mr. John Charlton, must have worked hard to get it finished. It would without doubt have been finer if he had been able to make haste more slowly. "The 22nd of January, 1901," shows a rugged old fisherman in Cornwall reading aloud to his family from a black-edged journal the news of the national misfortune, and is by Mr. Stanhope Forbes, whilst "God Save the King," by Mr. W. Hatherell, shows the York Herald proclaiming Edward VII. at Chancery Lane.

BUT it is in the large canvas by M. Benjamin Constant "Her Late Majesty Queen Victoria" that the greatest interest will naturally centre. The picture itself has a history apart from the fact that it represents our late Sovereign. It was the result of a vision which came to the artist one day in the House of Lords, and the face was copied from a small, very exact, likeness of the Queen's face. It was painted in the first place for the proprietor of an illustrated journal, and was primarily intended for reproduction. The Queen gave no sitting for it; in fact, till the work was practically complete, she never saw it, and then it was at her own request. Last year it was exhibited at the Paris Salon; this year it occupies the west wall of the principal room of the London Royal Academy by command of His Majesty the King, draped with purple and black, and framed in with palms. Though the Queen sits upon the throne of the Upper House with all the insignia of Royalty, there are no subjects gathered around her, she appears alone as the embodiment of sovereignty, whilst the sunlight streams down upon her from an unseen window illuminating the head and bust. As far as a likeness is concerned, many will complain that M. Constant has not succeeded in showing the strongly defined character, the royal dignity which were so apparent in the face of the Empress of India. But then M. Constant laboured under the great disadvantage of never seeing the Queen till too late to materially influence his brush, and no one will deny that the whole conception has a kind of grandeur of its own, nor fail to turn towards No. 149 with quickened steps as they near the large room.

FEW nurses will disagree with me when I assert that for utter weariness of the flesh there is no panacea equal to a blow on the water. The change of surroundings, the fresh air, and the feeling that there is no need to do anything but sit still, all have a soothing effect, and many a nurse who leaves her ward certain that life is not worth living may return after a river ride quite bright and cheerful once more. For the benefit of nurses in general, more especially those whose hospitals are situated near dear old Father Thames, I may mention that after a brilliant inauguration with a procession of 36 steamboats on the first of May, the summer service on the river has begun. Substantial improvements have been made; all the steamers have been repainted; there is a ten minutes' service, and the fares between London Bridge and Battersea are only 2d. for the whole distance of 4¾ miles, and 1d. for intermediate stages to and from Nine Elms. There is the usual half-hourly service between Chelsea Pier and Kew Gardens, and also from Westminster to Greenwich and Woolwich. During the summer months a boat will run daily to and from Herne Bay, and another to Hampton Court and back for 1s. 6d. The new Palace steamers start a little later. *Royal Sovereign* and *Koh-i-noor* will sail to Southend, Margate and Ramsgate, commencing on the 25th of May, and *La Marguerite* will start her trips to Margate, Boulogne, and Ostend on June 26th. There is thus plenty of choice for all, and everything possible in the way of comfort and convenience is promised for the passengers.

Do you happen to have a friend who would like to act as a "fashion model?" I have a very small idea of the duties of such an occupation, but perhaps this advertisement which was taken from a daily paper may throw some light upon the subject:—

YOUNG lady REQUIRED, by a lady, as COMPANION and FASHION MODEL. Good references essential. Must have very small waist, about 16in. Comfortable home and salary £50. State correct age, height, and full particulars, to ——.

The salary offered is a fairly good one if the home is really comfortable, and the duties light, but then ladies with waists about 16 inches are not to be found every day in the week, and such trifles as displaced organs and impeded circulation deserve good remuneration. I take it for granted that no nurses are likely to apply. It seems rather unkind that the advertiser should afford no inkling as to the height she desires, because it would have prevented the application of several ladies whose inches round might be quite correct, but who possessed too many or too few when measured upwards.

### The Nurses' Co-operation.

WE are informed that the nurses have decided to demand a meeting both of the nurses attached to the Co-operation, and also of the members, before the end of the present month. An effort has been made to prevent the nurses from taking independent action by exhorting them not to be disloyal to the committee. If there has been disloyalty, it is on the part of a committee which was appointed to safeguard the nurses' interests and which is now upon its trial, because a few members who run its policy are charged with disloyalty to the whole body of nurses, whose interests they have neglected to safeguard by riding hobbies of their own. In a recent article (see our issue of March 16th, 1901, MIRROR, p. 310), we pointed out that the nurses' hard-earned savings had been sunk in a home which was not paying, and that unless it were made to pay it might land the Nurses' Co-operation in financial disaster. How serious the position is will be understood when it is realised that not only does the home not pay, but the committee have pledged the credit of the Nurses' Co-operation to the extent of £1,500 for a term of years.

It is quite clear that the only way to effectually protect the interests of the nurses by making the Co-operation do what it was established to accomplish is to call for the resignation of the whole of the committee, and to substitute for the present members those who will make it their whole duty to represent the interests and wishes of the nurses, for whose protection the society was incorporated. We hope, therefore, that the whole body of nurses in the Co-operation will act together, that they will form a small committee to carry out the wishes of the nurse-members of the Co-operation, and that the policy and action of that committee will be supported throughout without a dissentient voice. Unity is strength, and as the Nurses' Co-operation has done much already to secure to nurses their own, it is essential that those nurses who have benefited by becoming members of it should now sink all minor differences and determine to act together as one united whole.

In order to remove the want of information respecting their Co-operation from which we learn some of the nurses suffer, we have been asked to state briefly the history of the Association. The Co-operation was founded in 1891 by Mr. Capel Slaughter, Sir Henry Burdett, Mr. Charles Cheston, and the late Mr. Walter Burns (Chairman of the R.N. Pension Fund for Nurses). These gentlemen gave ungrudgingly of their time and brains to secure for nurses a safe and adequate return for their services. They also, with the co-operation of several nurses, guaranteed the necessary funds to establish the Association, and other friends of nurses joined the movement. They triumphed over a good deal of opposition, believing that such a society would prove of immense benefit both to nurses and the public, and that it could be carried on with financial success. The great success of the venture under Miss Phillipa Hick's able pilotage fully justified their belief. Having seen the Co-operation upon a financially sound basis, and prosperous in every respect, the founders ceased to take any active part in its management. Since then the management has been "captured" by a small section of the committee, the nurses have grown to know less and less of their Association, and they have now a liability per annum to support the Howard de Walden Home, which does not meet the wants of the majority, and which is carried on at a loss. Finally the committee have lost for the nurses the able services of a superintendent who had their full confidence.

Nurses do not always understand figures, and it seems necessary for the complete understanding of affairs at the Co-operation to explain that the contribution of 2s. 6d. per annum to the Home cannot be regarded as the only contribution the nurses make towards it, if their earnings, as they do, go towards paying the deficit which occurs on its accounts, as under the present management the Home is losing.

### Florence Nightingale.

BORN MAY 12, 1820.

THERE lives the sweetest woman yet
That ever soothed the aching brain,
Or donned an angel's amulet
To stay the piteous pangs of pain.
She, who with zeal and saintly vow,
Went out to succour 'mid the fray,
Sits, with her wan and crownless brow,
Bent down with weight of years to-day.

For her the wounded soldiers prayed,
And kissed her shadow as she passed:
Her gentle presence pain allayed,
And dimmed the neighbouring battle-blast.
All England rang with pride and praise
Of her, whose life was lent to save,
Who, through those dark and bloody days,
On mercy's mission, dared the grave.

But now, alas! her step is slow,
Her eye, once bright, too faintly beams;
Yet do the days of long ago
Come back to fill her latest dreams.
Her happy heart grows young again,
Her soul enjoys, in calm content,
That higher bliss the few attain—
The conscience of a life well spent.

Sweet Nightingale, long lost to sight!
(How like our dulcet bird of May,
That only looms in England's night,
And sings till shadows melt away!)
We bring thee from thy native grove,
The weary twilight hours to leaven,
New wreaths of reverence and love—
Thy crown must be the gift of Heaven.

*Bernard Malcolm Ramsay.*

### Everybody's Opinion.

[Correspondence on all subjects is invited, but we cannot in any way be responsible for the opinions expressed by our correspondents. No communication can be entertained if the name and address of the correspondent is not given, as a guarantee of good faith but not necessarily for publication, or unless one side of the paper only is written on.]

### THE NURSES' CO-OPERATION.

"MISS MARY GOODIN" writes: In case someone with more ability than myself omits to correct a statement in this week's MIRROR, concerning the Nurses' Co-operation, I will make an attempt myself. The article states that the protest was signed by *more* than 400 of the 500 nurses on the staff. The signatures number only 350. If readers of your paper will refer to your comments on previous reports, I think they will find they have always been favourable; how then I would ask can this fact be reconciled with the following statement:—"The dissatisfaction of many years has come to a head?" If you allude to internal dissatisfaction it is the first I have heard of it, and I have been a worker on the staff since 1894. As a nurse representative elected by the nurses, I have attended a number of committee meetings, and have always had a free voice. [The above is a distinct mis-statement. The exact number of the signatures to the petition which reached us was 401.—ED. HOSPITAL.]

"NURSE E. B." writes: As there is likely to be some controversy "about the Nurses Co-op.," I would suggest that in justice to the matron, committee, and nurses themselves, your very widely read paper should decline to publish anonymous letters about it. "Aprons" writes a disloyal letter in your last week's issue, adding she "knows little of the Co-operation now" as she "has her own connection, and only continues to belong to the Co-op. from feelings of loyalty!" It would be as well if such nurses abandoned the Association altogether, instead of writing things against it to which they are ashamed of putting their names. There are dozens and dozens of nurses out of the 510 on the Co-op. who have means of their own, and prefer to keep their own rooms all the year round to herding with a crowd of nurses in a large home, and yet do not object to pay the small half-yearly sum of 2s. 6d. towards its maintenance until the day comes when, like the Girls' Friendly Home near by in Great Portland Street, it is self-supporting. We nurses cannot grumble at this exorbitant tax, as it is the only subscription we are asked for during the year. The Nurses' Co.-op. has flourished ever since it was founded in 1891, and the management had better not be left to people like "Aprons," who owns she does not know how to set about it.

"Mr. P. Michelli, secretary of the Seamen's Hospital Society," writes: As one of the original members of the Nurses' Co-operation and one who took an active part in the establishment of the Institution and the constitution of its committee, it is with regret that I read in your paper that so flourishing a corporation should have fallen upon troublous times. During the whole of my connection with the Co-operation I was an ardent advocate for the management being vested in the nurses themselves, for I considered, and I consider still, that a body of women such as those I knew when the Co-operation was founded were perfectly capable of managing their own affairs. The rock upon which the Co-operation has struck is "clique." I saw this difficulty, but was willing to trust to the good sense of the majority of the nurses; but I see that I was mistaken, and that probably the original founders lost control of the Co-operation too soon. I am not blind to the fact emphasised by so many nurses that the particular nature of their calling sometimes prevents them taking an active part in the selection of their representatives, but I am confident that they might do more to see that they are properly represented. There are most of the original members of the Co-operation still in London, and I doubt not that they would be willing to take up the work where they left it and give the Co-operation a new start. It is important that every member of the committee of management should have a knowledge of nurses and their requirements.

### REIGATE ISOLATION HOSPITAL.

"An Interested Onlooker" writes: The Borough of Reigate has been most unfortunate in its Isolation Hospital. Some years ago a temporary building was erected and a man and his wife were placed in charge—the man to act as porter, the wife as matron. Things went on satisfactorily for some time until a sanitary inspector was appointed manager. Then began friction. Matrons changed; and no wonder, considering that the sanitary inspector was in the habit of telling them what to order for housekeeping and how to do so. When the new Isolation Hospital was built the sanitary inspector was informed by the Council that he had nothing to do with the place; but he thought otherwise, and still continued to act as though he was manager. At last the old hospital was reduced to the necessity of having a woman who had been in the habit of coming to do a day's washing left to take charge of the patients. I have no doubt it would be very difficult to get a nurse to stay under the management of a sanitary inspector. Then a matron was appointed and took up duty, but she also had trouble from the same source as the others. I wonder if there is another hospital in the United Kingdom where the doctors notify their cases to the sanitary inspector, where he arranges about discharging patients from hospital, and where an inspector is put over the doctor and matron. Perhaps some of your readers may be able to tell me. Now the hospital is as bad as ever. The matron left through illness, and I am afraid it will be a difficult matter to get a matron if things are not greatly altered. I have always understood that hospitals were managed by a hospital committee and the doctor.

### CROYDON UNION INFIRMARY.

"Deeply Interested" writes: Just one question as I have no wish to enter into a controversy: "On what grounds does Fair 'Treatment' consider that Miss Julian is unsuitable for the position of matron?" By the same method of reasoning by which she concludes that "Justice" and myself are ladies of Miss Julian's type, and that it is one word for her and two for ourselves? If so the inference is obvious.

"A Woman of no Importance" writes: I have followed with much interest the correspondence that has been published lately with regard to the matron and nursing staff at the Croydon Infirmary. Firstly, I think some will side with me in thinking the nurses have received a great injustice insomuch as after working three years for their certificate—not a short period—a matron should refuse to sign it, "because they were not suitable women." Should she not have known at the end of three months whether they were suitable or not? not to have allowed the full time of training to elapse, and then to place objections. Secondly, if those nurses were not suitable women, why were they permitted to go on with the work and no report made as to their unfitness? If they had been told at the commencement they might have tried elsewhere. A Poor Law infirmary is the most likely place (if a nurse is unfitted for her work) for that unfitness to be found out before the end of three years. I think the class of patients especially are the most trying one can nurse—being mostly chronic and permanent. They have great opportunities of finding fault with those who come in contact with them, and as a rule they do not mince matters. Thirdly, there are women acting as matrons and superintendents who are no more capable of holding those appointments than they are capable of flying. I once had a very bitter experience. After six months' work I was dismissed. Upon asking the reason I was told by the matron that there were "two or three little things I had done which were not her way of doing; and my way did not come up to her standard of nursing." I did not mind leaving under those circumstances, but when I went to that matron I had a good testimonial and she refused to give me one. So that I can fully sympathise with those unfortunate nurses who were similarly treated. With those in authority I have much sympathy; unless they are especially adapted it is hard work, but when not so they should not be in office. And there should be a means of clearing them out, and the sooner they are dispensed with the better for those who do the work. Lastly, it is bad in every sense for nurses to run to the matron with every little grievance, as some do. Matrons, sisters, and nurses should each concede to each other their place. But one cannot expect perfection. Justice one might have and naturally expects if one tries to do one's duty.

### A CHURCHWOMAN'S PROTEST.

"A London Nurse" writes: Like many other people I am sometimes seized with a desire to hear a popular preacher, and on Sunday week, accompanied by a friend, I went to St. Peter's, Vere Street. On arriving we were told by one of the vergers (not too politely) that there was no chance of our getting seats in the body of the building, so we went into the gallery. There were a number of unoccupied seats—as there were also down below—but we were informed that we might not take any, even though the service had proceeded to the Psalms. This I call a flagrant piece of injustice. One can understand the custom of retaining seats for seatholders or for regular worshippers until the commencement of the service, and one does not mind being turned away because there is not a vacant seat, but to stand to the verge of weariness and still see unoccupied seats, or worse still to see two people turned out of a pew at the very back of the gallery by three females who chose to be late instead of being in their places—at any rate by the time the bell ceased—was too much, and my friend and I left the church feeling sore and angry too. Unfortunately we cannot claim to be "aggrieved parishioners." In many churches the rule is—"all seats free after the service has commenced." Why not at St. Peter's, Vere Street? I may add that I was not the only nurse who left the building after weary waiting.

### A Book and its Story.

### THE LIFE OF THE GREAT QUEEN.*

QUEEN VICTORIA'S life, by Mr. Holmes, in its present form is a reprint of the more sumptuous edition published in 1897, minus the illustrations and the references to them, which appeared then. In addition a brief record of the leading events in the intervening three years, 1897–1901, brings the narrative down to the closing moments of the Queen's life. There has been little delay, wisely, in bringing out this latest contribution to the literature, authentic and otherwise, which has flooded the country since our beloved and revered sovereign passed to her rest. Mr. Holmes's "Life" has a special claim to attention as being that "authorised by Her Majesty Queen Victoria." The Queen's librarian would naturally have unusual opportunities for the collection of facts connected with her life, and he has been greatly aided in the making of his book by notes graciously supplied personally by Her Majesty, and in the correction of many little fables which have grown up around much that has been written. Originally the work was undertaken by the request of the publishers, Messrs. Boussod, Valadon and Co., who recognised the value of an edition in which the story could be told mainly by the aid of illustrations from pictures in the royal collections. Since the Queen's death a cheaper popular edition which should place the book within the reach of a wider public has naturally been called for. Mr. Holmes begins his story by tracing the Queen's ancestry back to Henry VIII., and devotes his first chapter to bringing her lineage down to her Royal father, the Duke of Kent. It is a singular fact that Queen Victoria alone, out of the three Queens who preceded her, stands in the unique position among the Queen Regents of Great Britain "as being the only one who has been blessed with direct heirs." No one can read the narrative of her private as well as public life unmoved. The most callous must be struck by the sweet yet strong humanity which underlies every sentence of her contributions to the present work. "Like all great souls in her simplicity sublime"—with apologies to Lord Tennyson.

To go back to the beginning of her reign, although the story of the coronation has been told a thousand times, yet the touching theme is one that can never be other than irresistibly attractive. There is something so uniquely suggestive in the thought of that slight girlish form sitting alone on the throne as her loyal subjects advanced to offer their homage—archbishops, bishops, peers—in these quaint words, "I do become your liegemen of life and limb, and of earthly worship: and faith and truth I will bear unto you, to live and to die, against all manner of folks—so help me God." As she passed with the queenly dignity which always characterised her movements, through the brilliant throng on her way from the Abbey, "wearing her crown, her right hand holding the sceptre with the cross, and her left supporting the orb—which she found very heavy—followed by the peers and peeresses now wearing their coronets, the brilliant afternoon sun pouring through the windows made the scene one of incomparable splendour." Yes! we have heard it all before, but it is an old story, ever new, which, long as the Empire lasts, will find a sympathetic echo in the hearts of loyal subjects. Who can read the following tribute, written after the coronation from the pen of a contemporary writer, Mrs. Jamieson, with dry eyes?—words which could never have been written of any than one who was before all things a *woman*, and a sovereign: "She returned looking pale and tremulous, crowned, and holding her sceptre in a manner and attitude which said 'I have it, and none shall wrest it from me.' Even the much-abused philosopher, Carlyle, could not suppress his emotion at the sight, and was heard to mutter 'a blessing on her head.'" Here the author takes the opportunity of correcting an inaccuracy which has appeared in the statements about the number of guests entertained by Her Majesty after her coronation at the banquet in the Palace. "The only guests present were those staying in the house, among whom were Her Majesty's half-brother and sister, and her future father-in-law, the Duke of Saxe-Coburg. The crowd was too great indeed for anyone to have come, had they been invited." It is curious to read among other public festivities of "a three days' fair being held in Hyde Park . . . The conduct of the crowds being excellent."

Of the ordinary routine of the Queen's day in early years an account is given by which it is seen that every moment was occupied. Business first, and then pleasure. "At two she rides with a large suite (and she likes to have it numerous) . . . After riding she amuses herself for the rest of the afternoon with music and singing, playing and romping with the children, if there are any in the castle—she is so fond of them that she generally contrives to have some there—or in any other way she fancies." And then follows the account of more stately functions closing with "at half-past eleven her Majesty goes to bed." "The Queen orders and regulates everything herself. She knows where everybody is lodged in the castle, settles about the riding and driving, and enters into every particular with minute attention." We have all heard "how this regularity in the allotment of time, and careful attention to every point of detail, both in her own household and the discharge of public duty, has been one of the marked characteristics of Her Majesty's life throughout the whole of her long reign."

"Fierce is the light that beats upon the throne." But no light, however searching, could reveal flaws in the ideal life which the Queen conceived in her girlhood, matured matronhood, and brought to perfection in later years.

Queen Victoria was a woman of many sorrows, and, like others to whom warm affections have been given, was called upon to bear the burden of bereavements frequently. Her tender heart went out naturally to all who were suffering, and her own sorrows made her able to sympathise deeply with all who were afflicted. She fully realised the meaning of the word *sympathy*—viz., that of not merely feeling for, but *with* the sufferer.

There was perhaps no class for whom her kind heart ached more often than for her soldiers and sailors; in the numerous extracts from her diary which are given we have glimpses of this. Here is one, after her distribution of medals to the wounded officers and men after the Crimea. It comes at a time when it appeals with special force to the nation: "Noble fellows! I own I feel as if they were my own children; my heart beats for them as for my nearest and dearest! They were so touched, so pleased, many I hear cried; and they won't hear of giving up their medals to have their names engraved upon them, for fear they should not receive the identical one put into their hands by me!" One young officer, Sir Thomas Trowbridge, who had lost one leg and the foot of another was dragged up in a Bath chair to receive his decoration. Upon the Queen saying she would make him one of her aides-de-camp, he replied, "I am amply repaid for everything," she adds, "One must love and revere such soldiers as these."

Mr. Holmes concludes his very useful and workmanlike record of Queen Victoria's life and reign with the words: "Of the life of the Queen and the great and widespread influence she exercised over the history of her own and other peoples, no less by the purity of her life than by the integrity and wisdom she brought to bear on the conduct of affairs, the story has yet to be written." In the meantime his own record—with valuable contributions by her Majesty, Sir J. Martin, and others—will fill very creditably the gap until a more intimate and exhaustive one appears.

* "Queen Victoria." By Richard R. Holmes, M.V.O., F.S.A. (Librarian at Windsor Castle.) Publishers: Longmans, London. Price 5s. net.

### For Reading to the Sick.

PATIENCE.

Thy work this hour is Patience!—If the Past
Hath set its image there where naught decays,
Deny not its own work to this thy last.
Strong yearnings ever mark'd thy vanished days,
And outstretch'd longings after absent ways;
That all is past; and now thy heart incline
To seize the present good as by it strays!
To Heaven's all-gracious Will thyself resign!—
The Heavenly kingdom this; and this is Life Divine!
*Williams.*

Nearer, my God to Thee!—Nearer to Thee!
E'en though it be a Cross that raiseth me,
Still all my song shall be—Nearer my God to Thee!
Nearer to Thee! *Adams.*

Holy Scripture bids us "run with patience the race that is set before us."

One might have anticipated that energy or zeal would be the word, rather than patience; but no, it is patience.

If not even a race, then surely nothing that appertains to duty should be done in mere hurry or depend upon impulse. Our race is for life or death, yet must it be run peacefully.

One element of excitement is far removed from it. A race it is, yet only to attain a goal, not to outstrip competitors. On the contrary, there is scarcely a greater help to one's own running than to lend a hand to a halting brother or sister.

Our mighty Forerunner Whom no saint follows except at an infinite distance, even Christ Who in all things hath the pre-eminence, ran His mortal race with glory as of the sun, and alacrity as of a bridegroom, and strength as of a giant: nevertheless He ran it with such peace and patience that He vouchsafed to become as one who carries lambs in his bosom and gently leads the feeble ones.

As of yore to our forefathers, so to-day He saith to us: "Come unto Me, all ye that labour and are heavy laden, and I will give you rest. Take My yoke upon you." Now a yoke is not for standing still, but for toiling forward: and thus He promises us rest while we toil, even as He requires of us patience while we run our race.—*Christina Rosetti.*

The Land beyond the Sea!
When will life's task be o'er?
When shall we reach that soft blue shore,
O'er the dark strait whose billows foam and roar?
When shall we come to thee,
Calm Land beyond the Sea?

The Land beyond the Sea!
How close it often seems,
When flushed with evening's peaceful gleams;
And the wistful heart looks o'er the strait, and dreams!
It longs to fly to thee,
Calm Land beyond the Sea!

The Land beyond the Sea!
Sometimes distinct and near
It grows upon the eye and ear,
And the gulf narrows to a thread-like mere;
We seem halfway to thee,
Calm Land beyond the Sea!

The Land beyond the Sea!
Sweet is thine endless rest,
But sweeter far that Father's Breast
Upon thy shores eternally possest;
For Jesus reigns o'er thee,
Calm Land beyond the Sea!
*Rev. F. W. Faber, D.D. (abridged).*

### Notes and Queries.

**The Editor is always willing to answer in this column, without any fee, all reasonable questions, as soon as possible.**
**But the following rules must be carefully observed:—**
1. **Every communication must be accompanied by the name and address of the writer.**
2. **The question must always bear upon nursing, directly or indirectly.**

**If an answer is required by letter a fee of half-a-crown must be enclosed with the note containing the inquiry.**

*Instep Support.*

(54) Can you give me the address of the shoemaker (in Norwich, I believe) who makes supports for flat feet?—*A. F. O.*

Do you refer to "Pond's instep supports," advertised some time ago in Mirror? Messrs. Gorchs, 76 and 77 Brompton Road, W., also supply them.

*Address.*

(55) Kindly tell me if there is a hospital for infectious diseases in the Isle of Wight, and how I can obtain the address.—*R. L.*

There is a small cottage hospital near Ventnor. Apply for particulars to the Secretary of Undercliff Hospital Committee.

*Phthisis.*

(56) Will you kindly mention a home suitable for a servant with phthisis? The case is somewhat advanced, but it is considered suitable for open-air treatment.—*A. C. S.*

Most of the hospitals for consumption have adopted, in a greater or less degree, the open-air method of treatment. A governor's letter would admit the patient free to the North London Hospital for Consumption and Diseases of the Chest, Mount Vernon, Hampstead, N.W.

*Bed Pull.*

(57) Kindly tell me if there is any apparatus by which a heavy paralysed patient can lift herself or be lifted easily in bed.—*M. M. D.*

What is known as the "Gorham bed" is designed for such cases. Any firms advertising in The Mirror would send particulars of pulls to fix on to beds, whilst if cost is important a bar suspended from a crook in ceiling is effectual.

*Home for Incurables.*

(58) Would you kindly tell me of a home in Scotland suitable for a lady suffering from an incurable internal disease? She could afford to contribute a little towards her maintenance.—*Nurse P.*

Apply for particulars to the Aberdeen Hospital for Incurables, Office, 42 Union Street, Aberdeen; The Longmore Hospital for Incurables, Salisbury Place, Edinburgh; the Gleniffer Home for Treatment of Diseases considered Incurable, Paisley; and the Hillside Home for the Treatment and Relief of Incurables in Perth and Perthshire, Perth.

*Germany.*

(59) Can you kindly tell me if there are any English private nursing institutions in Germany? If there are, would you give me the addresses?
*Nurse Emily.*

It is very difficult to obtain reliable particulars of private institutions, more especially of those abroad. You might refer to the German Consul, 49 Finsbury Square, E.C.

*Number of Staff.*

(60) Kindly tell me the number of nurses there should be on the staff of a hospital of twelve beds.—*Ignor.*

It entirely depends upon the amount of work required and the class of cases treated.

*Dilemma.*

(61) Our committee is anxious to keep the work (district) within the limits of the present staff; when, therefore, we were requested to remove a typhoid case (who was not our patient) to the fever hospital, I refused to do so on this account, and also because the hospital had nurses of its own available. My conduct has been criticised in an anonymous letter to the local press, and I should like to know if I acted rightly in the matter.

It depends entirely upon who requested you to remove the patient. If it were someone who was authorised to do so, you should have obeyed first and protested afterwards. On the other hand, a district nurse can only take orders from those deputed to issue them.

*Lupus.*

(62) Will you please tell me which hospital in London gives the new treatment for lupus? How can I gain admission?—*Miss D.*

Write to the Secretary, the London Hospital, E.

*Travelling Nurse.*

(63) Do you think I should be able to obtain a position as travelling nurse to a private patient? I have had three years' training, but I do not know any foreign language.—*Nurse Catherine.*

You could always try and hear of a patient, either privately or by advertisement. It is, of course, desirable to speak foreign languages, but this accomplishment is not always necessary.

*District Nurses.*

(64) What training institutions, if any, supply district nurses?—*E. R.*

The Queen Victoria Jubilee Institute for Nurses; St. Katherine's Precincts, Gloucester Gate, Regent's Park, N.W. Apply the General Superintendent.

**Standard Books of Reference.**

**"The Nursing Profession: How and Where to Train." 2s. net; post free 2s. 4d.**
**"The Nurses' Dictionary of Medical Terms." 2s.**
**"Burdett's Series of Nursing Text-Books." 1s. each.**
**"A Handbook for Nurses." (Illustrated.) 5s.**
**"Nursing: Its Theory and Practice." New Edition. 3s. 6d.**
**"Helps in Sickness and to Health." Fifteenth Thousand. 5s.**
**"The Physiological Feeding of Infants." 1s.**
**"The Physiological Nursery Chart." 1s.; post free, 1s. 3d.**
**"Hospital Expenditure: The Commissariat." 2s. 6d.**
**All these are published by The Scientific Press, Ltd., and may be obtained through any bookseller or direct from the publishers, 28 and 29 Southampton Street, London, W.C.**

### Travel Notes.

By Our Travelling Correspondent.

### LXXI.—THE BORGIAS IN ROME.

This famous Spanish family, who became powerful in Rome after the elevation of Calixtus III. to the Papal throne in 1455, has inspired the world with a lurid interest in their sinful lives. Time often whitewashes many historic sinners, as real facts come to the surface, separated from the effects of the angry party and personal hatreds which surrounded them in their lifetime. So to a great degree is it with the Borgias. Undoubtedly Rodrigo, afterwards Alexander VI., was an evil man, though in many ways a powerful and wise ruler. We cannot acquit him of boundless ambition and the determination to aggrandise his family, no matter at what cost, nor is it possible to shelter his memory from the overwhelming charges of profligacy and immorality which have cast so sinister a shadow upon it. Late research, however, seems fortunately to have proved his innocence of some of the crimes long attributed to him, and that a confusion between his name and that of his son Cesare has unknowingly added to the reputed sins of both. Rodrigo was born in 1431, and became a soldier, with no thought of the priesthood until 1455, when it was forced upon him by his powerful relative Calixtus. This somewhat excuses (considering the spirit of the times) his connection with Rosa Vanozza—by some chroniclers said to be a member of the Farnese House. By him she had several children, among others Cesare and Lucrezia. He never severed his relationship with Rosa, and she lived in Rome, though ostensibly apart from him, till her death. On his behalf, for whom it is so difficult to say anything laudatory, one must admit that he seems to have been warm-hearted and tenderly attached to his children.

### The Truth about Lucrezia.

She first married the Lord of Pesaro; this marriage was dissolved, without reason given by the Pope, and has been the cause of the deepest stain on his name. Her second husband was the Duke of Biscaglia; he was murdered in 1500, two years after his marriage. A short time previously he had been stabbed by emissaries of Cesare's on the steps of St. Peter's; he recovered, however, and, carefully watched by his wife, who feared poison for him, he escaped till 1500, when he was strangled in his bed. The cause of this crime remains obscure. It seemed however sufficient that for some reason he was obnoxious to Cesare Borgia and therefore he was quietly put out of the world and no questions were asked. In those lawless times a murder more or less, especially by one belonging to so powerful and unscrupulous a family as the Borgias, passed with little comment; it was too dangerous to run the risk of incurring their anger. So when the unhappy Duke of Gandia was murdered and his body found in the Tiber, no one ventured to raise any agitation. It was known beyond doubt that his brother Cesare had ordered his assassination, but no steps were taken to bring the matter to light. Cesare was killed in the siege of Pampluna, and one feels that it was too honourable a death for one so mean, cruel and avaricious.

### Lucrezia as a Poisoner.

It seems conclusively proved now that the unhappy Lucrezia (unhappy because the offspring of sin and the child of so evil a man as Alexander VI.) was guiltless of the crimes ascribed to her as a wholesale poisoner. After the death of the Duke of Biscaglia she married Alfonso of Este and went to live at Ferrara. The Ferrarese show with much pride the window of the room in which she is supposed to have given one of her famous suppers, diversified with the assassination of one of the guests as well as the wholesale poisoning of the rest! In Rome a house is shown near to S. Pietro in Vincola, which the proud inhabitants also claim to have belonged to Lucrezia, and to have been the scene of the gifted toxicologist's little entertainments. In reality it is now proved that these accusations against poor Lucrezia, as well as those of other dark crimes, are purely imaginary, and the outcome of the hatred, fear, and almost superstitious horror with which the family inspired the Romans of their day.

### The Death of Alexander VI.

He died in 1503, being at that time about 74. There are many accounts of his death but all concur in the supposition that he was poisoned, and it is thought that the fatal dose had been prepared by his orders for another. This is Ranke's account of the matter—of course much condensed:

> The Cardinal having received a gracious intimation that the Pontiff together with the Duke of Valentinos designed to sup with him . . . and that his Holiness would bring the supper with him, the Cardinal suspected that this determination had been taken for the purpose of destroying his life by poison. . . . Considering of the means by which he might save himself . . . he sent in good time to the Pope's carver . . . who, when he had come to the said Cardinal, was taken by him into a secret place when the Cardinal showed the carver a sum of 10,000 ducats in gold, which he persuaded the carver to accept as a gift and to keep for the love of him. . . . The Cardinal offering moreover all the rest of his wealth—for he was a very rich cardinal—and declared to him "You know of a certainty what the nature of the Pope is and I know that he has resolved to procure my life by poison, through your hand"—wherefore he besought the carver to take pity on him and to give him his life. . . . Now the orders were that the carver should present three boxes of sweetmeats after supper . . . and in that for the Duke there was poison. . . . The said Cardinal gave directions to the carver in what manner he should serve them so as to cause that the box of poisoned confect should be placed before the Pope . . . who, trusting in his carver and seeing the Cardinal tasting, judged that no poison was there and ate of it heartily; while of the other, which the Pope thought was poisoned, but which was not, the Cardinal ate. Now at the hour accustomed, according to the quality of that poison, his Holiness began to feel its effect and so died thereof.

This took place in a house close to the old Porta Angelica, and there seems confirmation of the story of poison in the fact of the terribly quick decomposition of the corpse. No honours were paid to the memory of so hated a man; his body was thrown without any respect into a coffin and hastily buried in the crypt of St. Peter's, whence it was taken by the orders of his successor, Julius II., and interred, unwatched, unmourned, and unhonoured in the church of S. Maria di Monserrato. Ere the body was cold, the apartments which had been peculiarly his own in the Vatican were rifled by emissaries of Cesare's. A more hideous death and burial could hardly be imagined. Hare says, quoting from Guicciardini: "All Rome ran with indescribable gladness to visit the corpse. Men could not satiate their eyes with feeding on the carcase of the serpent who, by his unbounded ambition and pestiferous perfidy, by every demonstration of horrible cruelty, monstrous lust, and unheard of avarice, selling without distinction things sacred and profane had filled the world with venom."

### TRAVEL NOTES AND QUERIES.

Grindelwald in May and June (Yungfrau).—At last the early summer trips are published to Switzerland. Send twopence for postage and I will forward you all the information I have been able to glean.

